# Therapeutic Potential of IL-17-Mediated Signaling Pathway in Autoimmune Liver Diseases

**DOI:** 10.1155/2015/436450

**Published:** 2015-06-04

**Authors:** Haiyan Zhang, Francesca Bernuzzi, Ana Lleo, Xiong Ma, Pietro Invernizzi

**Affiliations:** ^1^Liver Unit and Center for Autoimmune Liver Diseases, Humanitas Clinical and Research Center, Via Manzoni 56, Rozzano, 20089 Milan, Italy; ^2^State Key Laboratory for Oncogenes and Related Genes, Key Laboratory of Gastroenterology and Hepatology, Ministry of Health, Division of Gastroenterology and Hepatology, Ren Ji Hospital, School of Medicine, Shanghai Jiao Tong University, Shanghai Cancer Institute, Shanghai Institute of Digestive Disease, 145 Middle Shandong Road, Shanghai 200001, China

## Abstract

Emerging evidence reveals that various cytokines and tissue microenvironments contribute to liver inflammation and autoimmunity, and IL-17 family is one of highlights acknowledged. Although the implication of IL-17 family in most common autoimmune diseases (such as psoriasis, inflammatory bowel disease, and rheumatoid arthritis) has been extensively characterized, the role of this critical family in pathophysiology of autoimmune liver diseases (AILD) still needs to be clarified. In the review, we look into the intriguing biology of IL-17 family and further dissect on the intricate role of IL-17-mediated pathway in AILD. Considering encouraging data from preclinical and clinical trials, IL-17 targeted therapy has shown promises in several certain autoimmune conditions. However, blocking IL-17-mediated pathway is just beginning, and more fully investigation and reflection are required. Taking together, targeting IL-17-mediated responses may open up new areas of potential clinical treatment for AILD.

## 1. Introduction

Liver is an immunological organ with unique properties of immune tolerance, which can lead to systemic immune tolerance. Many genetically susceptible individuals suffer from autoimmune liver diseases (AILD) while some unidentified environmental factors trigger the breach in immune tolerance resulting liver as a victim. However, the exact pathogenesis of AILD is still unknown. Autoimmune hepatitis (AIH), primary biliary cirrhosis (PBC), primary sclerosing cholangitis (PSC), and autoimmune sclerosing cholangitis are the major forms of AILD. Besides, a proportion of patients within the spectrum of AILD may present with overlapping features of two classical disorders such as AIH, PBC, and PSC. These patients are often referred to as “overlap syndromes” [1, 2].

AILD have fluctuating and progressive courses with alternating relapses and quiescences. The spectrum of AILD is diverse, ranging from insidious onset with abnormal liver function tests to fulminant hepatic failure. The causes of these clinical conditions are complex and most likely heterogeneous. The mechanisms responsible for the progression of AILD are yet to be fully clarified. However, recent studies have demonstrated that cytokines play a pivotal role in the induction of immune responses during the development and progression of liver diseases. Among them, IL-17 family is one of the dominant pathogenic components in autoimmune inflammatory diseases, such as multiple sclerosis (MS), psoriasis, and rheumatoid arthritis (RA) [3–5]. Of interest, its role in AILD still requires clarification. On this basis, this review addresses the current data regarding the roles of IL-17 signaling in the pathogenesis of AILD and provides new insight into therapeutic potential of targeting IL-17-mediated responses.

## 2. General Features of Interleukin-17

### 2.1. IL-17 Family (Discovery, Structure, Resource, and Function)

IL-17A (commonly referred to as IL-17) was first identified as a product of rodent activated T cells in 1993 and was initially known as cytotoxic T lymphocyte associated antigen 8 (CTLA-8) [6]. Since then, other five members of IL-17 family, IL-17B, IL-17C, IL-17D, IL-17E (also called IL-25), and IL-17F have been discovered based on homology in amino acid sequences [6–9].

IL-17A is the most well investigated member of the IL-17 family and acts on multiple cell types to enhance the production of various proinflammatory molecules including cytokines (such as TNF and IL-6), chemokines (such as CXCL2 and MCP-1), mucins acute phase proteins, and matrix metalloproteinases [10–16]. Overall, IL-17A exerts a wide range of functions in autoimmune diseases, host defense, transplantation, allergy, and malignancy [17–21].

With the family, IL-17F is the most homologous protein to IL-17A (~60%) and resembles IL-17A in both the cellular sources and regulation function [22]. Interestingly, IL-17A and IL-17F exist as homodimers [10]. In addition, IL-17A and IL-17F also form a heterodimeric cytokine (IL-17A/F) with intermediate signaling potency [23].

Th17, a subset of CD4^+^T cells named for their ability to preferentially produce IL-17, is recognized as the major producers of IL-17A and IL-17F [24]. In addition, several innate immune cell types are described as sources for IL-17, including *γδ*T-cells, natural killer (NK) cells, natural killer T (NKT) cells, dendritic cells (DCs), activated monocytes, mast cells, neutrophils, and lymph tissue inducer (LTi) cells [25–28]. Of note, IL-17A is found to be approximately 10–30 times more potent than IL-17F [29]. However, IL-17F and IL-17A have overlapping yet distinct proinflammatory roles in host immune and defense mechanisms, such as mucoepithelial bacterial infections, inflammatory responses, and allergic diseases [30, 31]. For example, genetic deletion of IL-17F resulted in reduced experimental colitis caused by dextran sulfate sodium, whereas deletion of IL-17A in mice developed more severe intestinal disease [32].

Unlike IL-17F, IL-17E (IL-25) is the most distant cytokine from IL-17A in IL-17 family. Recent studies show that IL-17E promotes Th2-mediated immune responses, particularly contributing to airway inflammation and allergic disease [33–35].

Far less is known about the cellular sources, receptors/target cells and biological functions of the three remaining other IL-17 family cytokines. IL-17B is expressed in multiple organs, including small intestine, pancreas, spinal cord, and stomach [36]. IL-17C was detected in keratinocytes and tracheal epithelial cells [37, 38]. Similar to IL-17B, IL-17D is also expressed in a variety of organs and tissues, including heart, pancreas, and adipose tissue [22]. Recent studies from both human and animal models suggest that these IL-17 family members might possess an important role in inflammatory disease, which highlights the need for further exploration. For instance, IL-17B was observed to participate in a murine model of collagen-induced arthritis whereas treatment with IL-17B neutralizing antibody can successfully suppress arthritis [39].

### 2.2. IL-17 Receptors (Discovery, Structure, Expression, and Signaling)

Interleukin-17RA (also known as IL17R) was the first receptor identified for IL-17A [40]. IL-17RA is expressed in considerable cell types, including endothelial cells, epithelial cells, fibroblasts, and myeloid cells. However, subsequent studies have demonstrated additional receptor members are required to form a functional receptor complex for IL-17 signaling [41]. The IL-17 receptor family represents a group of multimeric proteins that consists of four other molecules, IL-17RB (also known as IL17Rh1), IL-17RC (also known as IL17RL), IL-17RD, and IL-17RE.

Recent studies of IL-17RB revealed the expression in NKT cells, memory Th2 cells, macrophages, lung epithelial cells, airway smooth muscle cells, and endothelial cells [42–44]. IL-17RB can bind to both IL-17B and IL-17E in vitro and actively participate in Th2 immune responses via IL-17E–IL-17RB interaction, particularly in airway inflammation [33]. Besides, genetic and biochemical studies indicate IL-17RB binding to IL-17E recruits IL-17RA to the complex in mediating IL-17E function [45].

IL-17RC was initially identified based on sequence homology to IL-17RA [46]. IL-17RC was found to share expression with IL-17RA in multiple cells, including epithelial cells, macrophages, and fibroblasts, but its expression on myeloid cells is much lower [47]. Of note, the assembly of a heterodimeric complex between IL-17RA and IL-17RC is required for the biological signaling of IL-17A and IL-17F. Nevertheless, IL-17RC may have some functions independent of IL-17RA; for IL-17RC expression is elevated in some organs and tissues such as small intestine and lung where the expression of IL-17RA is absent [30].

IL-17RD and IL-17RE were also identified through database mining; however, little is currently known about these two orphan receptors. Studies have discovered the expression of IL-17RD in epithelial cells and endothelial cells. Moreover, IL-17RD can pair with IL-17RA to mediate IL-17A signaling while mutations of the IL-17RD cytoplasmic domain can suppress IL-17A function [48]. On one side, IL-17RE RNA could be detected in lung, testis, kidney, stomach, and intestine; on the other side, the cellular expression of IL-17RE is poorly understood. Further studies will be needed to address the significance of IL-17RE [49].

These single-pass receptors share minimal structural homology, including a single transmembrane domain, an extracellular-fibronectin III-like (FnIII) domain, and an intracellular SEF/IL-17R (SEFIR) domain [29, 50]. As IL17R proteins possess the conserved SEFIR domain, with IL17 family ligands binding, this intracellular domain can directly interact with adaptor protein, ACT1 (also known as TRAF3IP2) to initiate signaling cascades, augmenting various inflammatory molecules that participate in immune responses. For instance, with the help of both ACT1 and TNF receptor associated factor 6 (TRAF6), the binding of IL-17A to IL-17RA can activate nuclear factor-*κ*B (NF-*κ*B) signaling pathway [51]. Additionally, IL-17A can activate the CCAAT/enhancer binding proteins (C/EBPs)-C/EBP*β* and C/EBP*δ* to induce the transcription of inflammatory gene such as IL-6 [13]. By contrast, IL-17R-Act1-TRAF-NF-*κ*B pathway can be negatively regulated by the binding of TRAF3 to IL-17R, resulting in suppression of downstream signaling [52].

## 3. Pathological Roles of IL-17 in Autoimmune Liver Diseases

Before the recognition of the distinct Th17 population, it was considered that Th1, Th2, and B cells were the dominant cellular components of pathology in autoimmunity. However, with the deepening of the studies about IL-17 family, IL-17 has been strongly implicated in the pathogenesis of autoimmune diseases, such as psoriasis, inflammatory bowel disease (IBD), RA, and systemic lupus erythematosus (SLE) [70–73]. Nevertheless, robust evidence demonstrates that autoimmune liver diseases implicate the IL-17 pathway (Table 1).

### 3.1. Autoimmune Hepatitis

AIH is a chronic inflammatory liver condition of unknown etiology that is putatively initiated by the aberrant autoaggressive immunity against hepatocyte-specific autoantigens [57]. AIH is a progressive necroinflammatory disease characterized by elevated aminotransferase levels, hypergammaglobulinemia, circulating autoantibodies, and histological evidence of interface hepatitis [74]. The abnormal autoimmune reactions in AIH are believed to be orchestrated by self-antigenic peptide activated T cells that trigger the antibody-mediated cellular cytotoxicity and contribute to the pathogenesis of AIH [75].

Traditionally, AIH has been associated with dysregulations of both innate and adaptive immunity. Recently, IL-17 pathway has caught the attention of hepatologists and immunologists for IL-17 that is inimitably positioned at the interface of both types of immunity. A recent study has suggested that the serum levels of IL-17 and IL-23 were significantly higher in patients with AIH compared to patients with chronic hepatitis B (CHB) and healthy controls. Moreover, the frequency of circulating Th17 cells and the gene expression of IL-17 in the peripheral blood mononuclear cells (PBMC) of AIH patients were also demonstrated to be substantially elevated when detected by flow cytometry and real-time PCR [53, 54]. It is important to note that the number of Th17 cells and the gene expressions of IL-17 specific transcription nuclear factor- retinoic-acid-receptor-related orphan nuclear receptor gamma (ROR-*γ*t) also were significantly increased in the liver of AIH patients compared to those with CHB and healthy controls. Interestingly, hepatic Th17 cells in AIH patients are increased in a disease severity dependent manner. In addition, the expression of IL-17-related cytokines, that is, IL-23, IL-21, IL-1*β*, and IL-6, was significantly augmented in the liver of AIH patients [53].

In mouse models of experimental autoimmune hepatitis, congruent findings reported that the expression of IL-17 in the livers and sera of AIH mice were significantly elevated compared to the control mice. It is noteworthy that administration of IL-17A monoclonal neutralizing antibody could markedly reduce histological hepatic necrosis and serum ALT levels compared to controls [54]. These data suggest that IL-17 pathway is likely to be a prime part of AIH pathogenesis.

The mechanisms underlying the breakdown of self-tolerance leading to AIH have not been fully elucidated, though numerous lines of evidence indicate that numerical and functional regulatory T-cell (Tregs) defects may play a permissive pathogenic role [76–78]. Accordingly, the disrupted regulatory circuits of Tregs may account for the aberrant dysregulation of IL-17-mediated inflammatory responses. Within the Tregs group, CD39^pos^Tregs have been shown to display specific suppression over IL-17 immunity compared to their CD39^neg^ counterpart [79]. More recently, studies have reported that CD39^pos^Tregs not only are decreased in frequency but also exhibit limited adenosine triphosphate/adenosine diphosphate hydrolysis activity in AIH patients, which in turn fail to suppress the overactive IL-17 pathway by effector CD4^+^ T cells [55]. These observations imply that impairment of CD39^pos^Tregs in AIH, an inability to suppress IL-17 production and a propensity to convert to IL-17 producing effectors, may contribute to defected immunosuppression and autoimmune liver condition. Notably, Tregs and Th17 cells share a common progenitor, though their developmental pathways differ. Previous studies have reported that Tregs could be generated from CD25^−^ (ngTreg) cells in AIH patients, which suppress the immune responses less efficiently than the freshly isolated Tregs [80]. More importantly, studies also demonstrated that ngTregs from AIH patients presented the greater proportions of IL-17^+^ and ROR*γ* ^+^ cells than Tregs from healthy controls. Furthermore, taking advantage of several different approaches for the removal of IL-17 influence, ngTregs from patients with AIH managed to differentiate into functionally stable immunosuppressive cells [56]. These data highlighted the crucial effect of IL-17 pathway on defeated function of Treg in AIH and laid a solid foundation for immunotherapeutic strategies aiming at reestablishing immune tolerance through Treg infusion in combination with IL-17 abrogation.

### 3.2. Primary Biliary Cirrhosis

PBC is a chronic inflammatory autoimmune disease that mainly targets the cholangiocytes of the interlobular bile ducts in the liver [81]. The diagnosis of PBC is made when two of the three criteria are fulfilled, that is, presence of serologically PBC-specific autoantibodies such as anti-mitochondrial antibodies (AMA), abnormal changes in biochemical tests indicating cholestasis for longer than 6 months, and histologically chronic nonsuppurative destructive cholangitis followed by progressive bile duct loss [82]. There have been significant advances in the understanding of the immunobiology of PBC which is regarded as a classical T cell-mediated autoimmune disease. Intriguingly, various cytokines and tissue microenvironments were shown to exert crucial roles in the regulation and propagation of autoimmune inflammatory responses. Accumulating evidence in mouse models and clinical studies has clearly demonstrated the pivotal involvement of IL-17 signaling pathway in the pathogenesis of PBC.

It has been well described that not only the serum concentrations of IL-17 and IL-17-related cytokines such as IL-23, IL-1*β*, and IL-6 but also the mRNA expression levels of IL-17-mediated signaling pathway such as IL-23 p19, IL-23R, and IL-17 in PBMCs from PBC patients were significantly elevated compared to the healthy control [57, 58]. Consistent with the IL-17 cytokines profile, peripheral Th17 cell population and IL-17 specific ROR-*γ*t expression of PBC patients were increased markedly compared to patients with CHB. On the contrary, peripheral Treg cell population and the expression of Treg specific transcription nuclear factor FOXP3 were decreased dramatically in patients with PBC relative to CHB disease control [57]. These observations strongly imply that the Th17/Treg imbalance appears to exacerbate the breakdown of immune homeostasis in PBC.

One of the intriguing questions in PBC is that the autoimmune attack is organ specific and the immunological destruction is selective focus on small bile ducts. Consistent with this notion, researchers concern more about the role of IL-17 pathway in the liver of PBC patients. Indeed, several studies have revealed that PBC patients exhibit elevated levels of hepatic IL-17^+^ infiltrates as compared with healthy livers [59, 60]. Of interest, our recent results address the extensive IL-17-associated cytokines microenvironment specifically in the livers of PBC and non-PBC control liver disease patients. Our observations have demonstrated that Th17-related cytokines and their cognate receptors, that is IL-23p19, IL-23p40, IL-17, and IL-23R, were expressed by the inflammatory cells localized to the portal tracts, with the IL-17^+^ cells aggregated around interlobular bile ducts intensively. More importantly, Th17 skewing was more prominent in advanced PBC patients [61]. This direct association of Th17 skewing with disease severity uncovers the significance of the IL-23/Th17 pathway in the perpetuation of the so-called traditional IL-12/Th1-mediated immunopathology in PBC.

Of noted importance is that small biliary epithelial cells (BECs) are regarded as the target cells of the autoimmune destruction in PBC [83]. Thus, numerous studies aim to unravel the contribution of IL-17 pathway on the pathogenesis of cholangiopathy. Interestingly, BECs possess IL-17-receptors (IL-17RA and IL-17RC) and also Act1 (an essential adaptor protein in IL-17-mediated signaling). By taking advantage of the cultured human BECs, stimulation with IL-17 could induce the production of inflammatory cytokines (IL-6, IL-1*β*, and IL-23p19) and chemokines (CXCL1, CXCL2, CXCL3, CXCL6, CXCL8, CCL2, and CCL20) [60]. These evidences have indicated that periductal secreton of IL-17 facilitate the migration of various inflammatory cells including Th17 cells, which in turn aggravate the chronic cholangitis and bile duct damage in PBC. Moreover, a subsequent study demonstrated that Langerin^+^ Langerhans cells (LCs), which were dominantly scattered around or within biliary epithelial layers of the damaged bile ducts, served as periductal antigen-presenting cells in PBC [84]. IL-17 managed to upregulate the expression of macrophage inflammatory protein-3*α* (MIP-3*α*) in human BECs dramatically, while MIP-3*α* is the main chemokine for attracting and recruiting LCs [62]. Collectively, IL-17 pathway exerts as an unneglectable component of the periductal cytokine milieu and biliary innate immunity in PBC.

Detailed studies in several well-characterized murine models of PBC have further confirmed the importance of IL-17 signaling pathway in the development and progression of PBC. To begin with, IL-2 receptor *α* knockout mice, one of the identified murine models manifesting characteristic clinical features of human PBC, exhibits increased frequencies of IL-17 producing cells in the liver [63]. Similar to PBC patients, IL-2R*α* KO mice also demonstrate aggregative IL-17^+^ staining in diseased portal areas compared to control. Notably, splenic CD4^+^ T cells cocultured with liver nonparenchymal cells (NPCs) from wild-type mice could augment IL-17 production approximately 10-fold compared to T cells alone [59]. These data suggest a novel connection between the induction of IL-17 responses and unique liver environment mediated by liver NPCs and IL-17-related cytokine milieus in cases of liver autoimmunity.

Secondly, 2-octynoic acid-BSA-immunized mice that develop an intense inflammatory cholangitis with similarities to humans with PBC are another classical PBC animal model. In some unique gene-deleted mice immunized with 2OA-BSA, it is interesting to find that deletion of IL-17A and IL-22, instead of IL-17F could attenuate biliary damage and inflammation. Moreover, reduced levels of AMA were detected in IL-17A-knockout mice compared with controls [64]. These finding propose a potential therapeutic intervention of IL-17 pathway for PBC.

Additionally, dominant-negative TGF-*β* receptor II (dnTGF*β*RII) mice are also one of the well-established spontaneous models that exhibit histological features of PBC with the same AMA specificity [85]. It is important to note that considerable patients with PBC have detectable serum ANA directed primarily against gp210 and sp100. Upon further investigation, it was revealed that sera from dnTGF*β*RII mice contained antibodies against gp210 and sp100. However, mice with germline deletions of the genes encoding IL-17-related cytokines, that is, IL-12p40, IL-23p19, IL-17, IL-6, and TNF-*α*, had dramatically lower titres of anti-gp210 antibodies, but not anti-sp100 antibodies [65]. In fact, several lines of suggestive evidence mentioned that positive-anti-gp210 antibodies often predict a hepatic failure type of progression in PBC [86, 87]. Deletion of those IL17-related cytokines that led to significantly lower serum anti-gp210 titres suggests that Th17 cells appear to orchestrate anti-gp210 generation. Taken together, these observations provide a clue to further dissect the possible interaction between IL-17 inflammatory responses and the appearance of PBC-specific autoreactivity.

### 3.3. Primary Sclerosing Cholangitis

PSC is a rare chronic cholestatic liver disease characterized by diffuse inflammation, destruction, and fibrosis of multifocal bile ducts leading to strictures predominantly in large- and medium-sized ducts of the biliary tree [88, 89]. At present, the critical etiology or the conclusive pathogenic mechanisms underlying PSC remain unclear. Numerous hypotheses have emerged and it has been widely accepted that abnormality of hepatic immune response contribute to initiation and progression of fibro-obliterative cholangiopathy [90, 91]. Recent evidence has revealed that Th17 cells and its canonical cytokine IL-17 are served as critically important contributors of PSC pathogenesis.

It has recently been described the numbers of IL-17^+^ lymphocytes in liver were significantly increased in PSC patients compared to AIH control patients. Additionally, IL-17A-expressing T cells aggregated around damaged bile ducts and in areas of neoductular proliferation [66]. It is increasingly recognized that dysregulated responses to pathogen stimulation, especially bacteria and fungi, may be the proximal contributor to the immune activation in PSC [67, 92]. Of significance, PBMCs from PSC patients manifested significantly increased frequencies of Th17 cells after pathogen stimulation compared to healthy controls and cholestatic controls. Interestingly, stimulation with* Candida albicans*, which was shown to have a negative effect on progression of disease, gained the highest frequencies of Th17 cells than other pathogens in PSC patients [67]. Furthermore, Th17 induction were noted by the selective stimulation of Toll-like receptors (TLR) 5 and 7, indicating that the pattern recognition receptors signaling pathways may intimately be involved in this response [66]. These evidence points to the speculation that aberrant exposure to pathogens in bile potentially lead to a robust induction of IL-17 responses, which could initiate and then perpetuate portal and biliary inflammation in PSC.

By using several currently available animal models resembling some individual characteristic hallmarks of human PSC, researchers can therefore obtain a better knowledge of IL-17 pathway. Experimental biliary obstruction models, such as bile duct ligation (BDL), share the cardinal features of obstructive cholestasis and biliary fibrosis with human PSC [93, 94]. Consistent with the reports from PSC patients, both the gene levels of IL-17-related cytokines and receptors (IL-17 A, IL-17F, IL-17RA, and IL- 17RC) in the livers and sera concentration of circulating IL-17A in BDL mice were strongly upregulated compared with their sham control. More importantly, BDL-induced liver fibrosis was apparently inhibited in IL-17RA−/− mice compared with their wild-type counterparts, manifested by a sharp decrease of collagen deposition, *α*-SMA^+^ myofibroblasts number and mRNA expression of fibrogenic gene, and so forth [68]. Intriguingly, liver resident cells such as Kupffer cells and hepatic stellate cells (HSC) can also show strong expression of IL-17 cytokines and their receptors in addition to Th17. Furthermore, IL-17 could stimulate the production of TNF-*α* and TGF-*β* from Kupffer cells, which then regulate the HSC activation via the Stat-3 signaling pathway and in turn promote the differentiation of IL-17 expressing cells [68, 69]. To date, however, there is no ideal single animal model that can demonstrate that all the characteristic of PSC patients exists. Therefore, we will rather call for more different models to elucidate the particular immune role of IL17/IL17R signaling within the pathogenetic steps of PSC.

## 4. Therapeutic Potential of IL-17-Mediated Signaling Pathway in Autoimmune Liver Diseases

**Figure 1:**
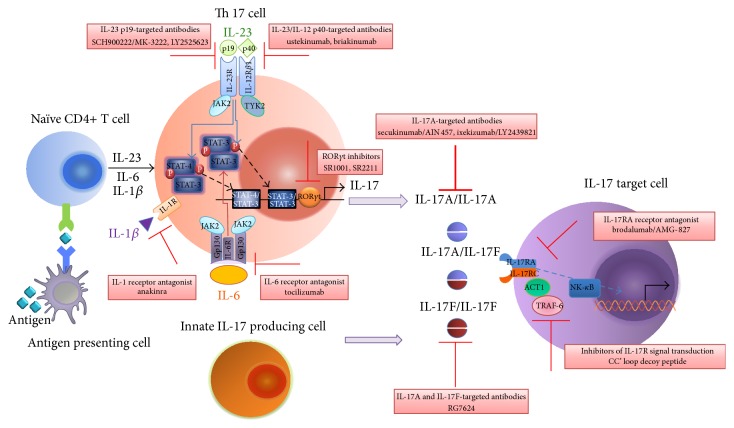
Therapeutic potential of targeting IL-17-mediated pathway. Several novel strategies in which members of the IL-17 family can be targeted are already in use for preclinical and clinical trials, including inhibitors blocking the differentiation of TH17 cells, IL-17 family cytokines or their cognate receptors-targeted antibodies and small molecule inhibitors blocking transduction of IL-17-mediated signaling in target cells.

As the studies mentioned above, excessive IL-17 responses have been proven to be a key mediator and potent drivers of liver-specific autoimmune diseases. With these comments in mind, targeting dysregulated IL-17 signaling pathway seems to be a new area of promising immunotherapy for AILD patients, especially for those patients who failed to respond to conventional therapy. However, detailed studies on the potential of adjusting violent IL-17 responses in AILD remain limited. On the other hand, it is also clear that pathological IL-17-mediated signaling is generally involved in many other human autoimmune conditions such as psoriasis, IBD, RA, SLE, and MS to name a few [3, 95–98]. Inspiringly, numerous clinical studies have examined the feasibility and effectiveness of reconstituting IL-17-mediated pathways including targeting IL-17-related cytokines/receptors, regulating the expression of IL-17 cytokines and IL-17/IL-17R downstream signaling for therapeutic purposes (Figure 1).

### 4.1. Blocking IL-17/IL-17 Receptors

Among different options for targeting IL-17 pathway, blocking IL-17 or IL-17R family, tend to be the most direct strategy. Thus, accumulating evidences stem from cellular, animal, and human studies uncovered the beneficial anti-inflammatory effect of blocking IL-17/IL-17 receptors on attenuating autoimmune diseases [99]. These encouraging results of targeting IL-17/IL-17R attract the attention of many medicine companies, and the predominant studies prefer to focus on IL-17A and IL-17RA. Blocking agents that neutralize IL-17A (e.g., secukinumab/AIN457, ixekizumab/LY2439821) and IL-17RA (e.g., brodalumab/AMG-827) are on track to Phase II/III clinical trials with satisfactory data. Taking treating psoriasis patients for example, all the published Phase II, randomized, double-blind, placebo-controlled studies results from these three humanized monoclonal antibodies offer encouragement, with the majority of (82% for secukinumab versus 9% for placebo, *P* < 0.001; 82.8% for ixekizumab versus 7.7% for placebo, *P* < 0.001; 86.3% for brodalumab versus 16.0% for placebo, *P* < 0.001) patients obtaining at least 75% improvement from baseline in the Psoriasis Area and Severity Index score (PASI 75) after 12 weeks of treatment [100–102]. Furthermore, Phase III results demonstrated that secukinumab not only significantly improved the clinical symptoms of psoriasis at 12 weeks but also offer faster yet longer efficacy than etanercept (TNF inhibitor) and placebo control [103]. Favorable findings were also reported in the clinical trial of secukinumab and ixekizumab with satisfactory relief of symptoms and safety in various IL-17-related autoimmune disease states, such as RA, ankylosing spondylitis, and noninfectious uveitis [104–107]. However, it is noteworthy that secukinumab is ineffective and has higher rates of adverse events for treating patients with moderate to severe Crohn's disease [108]. This unfavorable data alarm us to give more cautious consideration on IL-17 antibody before comprehensive clinical application.

Regarding targeting IL-17/IL-17R family, there are other members under the consideration. In the light of three different forms of IL-17 complex exist, that are IL-17A/IL-17A homodimer, IL-17F/IL-17F homodimer, and IL-17A/IL-17F IL-17A/IL-17A heterodimer. Researchers aim to target both IL-17A and IL-17F by using the same antibody RG7624 (Roche) to assess the efficacy and safety in the clinical trial. However, to the best of our knowledge, there have not been any published data from RG7624 Phase I trial. Concerning targeting IL-17 receptor, IL-17RC is believed to be another potential strategy in addition to IL-17RA. As IL-17RA and IL-17RC heterodimer function as the indispensable component of IL-17A and IL-17F signaling pathway [109], the therapeutic tool blocking both IL-17 RA and IL-17RC gains the attention. In concordance, previous study demonstrated that both IL-17RA and IL-17RC were implicated in the IL-17-triggered inflammatory cascade and blockade of both receptors by silencing interfering RNA or specific inhibitors was essentially needed to downregulate IL-17 pathogenic responses in RA [110]. Nevertheless, targeting IL-17RA may lead to undesirable effect on the IL-17E-mediated responses with IL-17RA/IL-17RB heterodimer as receptor for IL-17E [111]. In view of anti-inflammatory effect of IL-17E in inflammatory autoimmune disorders, thus targeting IL-17RC may be a more specific and efficient strategy.

### 4.2. Targeting Upstream of IL-17

New therapies inhibiting upstream of IL-17-mediated pathways have been proposed as attractive avenues of investigation. Several proinflammatory cytokines, IL-23, IL-6, and IL-1*β* in particular, are crucial for the development of IL-17-expressing cells and thus provoke the induction of IL-17 cytokines. Accordingly, clinical trials hold promise for targeting of these cytokines or their receptors as therapies of certain autoimmune diseases. First of all, neutralization antibody against the p40 chain of IL-23/IL-12 (ustekinumab and briakinumab) presented satisfactory efficacy and sustained safety in Phase II/III clinical trials for treating patients with active psoriatic arthritis or refractory Crohn's disease [112, 113]. More importantly, ustekinumab yielded desirable long-term safety in patients with moderate-to-severe psoriasis up to 5 years of follow-up in large approved Phase III trials [114, 115]. Given the fact that the p40 subunit is shared by IL-23 and IL-12, antibodies (SCH900222/MK-3222 and LY2525623) aiming at IL-23 unique subunit p19 should be more specific. Presently, the clinical trials of these two antibodies are still in process [116].

Secondly, IL-6 also participates in the induction of Th17 cells which are major source of IL-17A and IL-17F. Antagonistical antibody against the interleukin 6 receptor (tocilizumab) demonstrates significant clinical efficacy and satisfying safety; hence tocilizumab has been approved in many countries for the treatment of certain inflammatory autoimmune diseases such as RA and juvenile idiopathic arthritis (JIA) [117, 118]. In addition, IL-1*β* is another crucial cytokine for IL-17 induction. Of significant, IL-1 receptor antagonist (anakinra) has emerged as a promising clinical treatment for RA [119].

It has been widely accepted that ROR*γ*t serve as the master regulator for Th17 differentiation. Therefore, this renders us a clue that ROR*γ*t has the potential to be a promising therapeutic target for autoimmune disease. Actually, ROR*γ*t-specific inhibitors are on the way to become one of the most competitive biological drugs among all therapeutic fields. Emerging studies have focus on the drug target with several favorable candidates for further development [120–122]. Of relevance, SR1001, a high-affinity dual ROR*α* and ROR*γ* inverse agonist has been proved to effectively suppress the clinical severity of experimental autoimmune encephalomyelitis (a well-characterized model of multiple sclerosis) through its inhibition on TH17 cell differentiation [123]. It is noteworthy that ROR*α* and ROR*γ* exert extra functions outside of the immune system and are the important modulators of hepatic metabolism. As ROR*α* is the positive regulator of hepatic metabolic gene such as Rev-erb*α*, PAI-1, and Cyp7b1, administration of SR1001 may have broad implications in metabolic homeostasis [123–126]. Therefore, new inhibitors that are more specific to ROR*γ*t should be thoughtfully selected before further usage in autoimmune liver disease treatment. In concordance, SR2211, a potent ROR*γ* modulator which demonstrate selectively inhibitory effect on the production of IL-17 in cells, is seen as a potentially viable approach for treating autoimmune disorders [127].

### 4.3. Targeting Downstream Signaling of IL-17

Downstream targets of IL-17/IL-17R initiated signaling pathways are also promising candidates to inhibit their proinflammatory activity. Of note, the genetic variation of the Act1 gene (TRAF3IP2), which is the key adaptor to propagate IL-17-mediated downstream signaling, is associated with susceptibility to psoriasis and psoriatic arthritis by genome-wide association studies (GWAS) [128, 129]. As a first step to target Act1 for therapeutic strategies, recent study reported that blocking the interaction between Act1 and IL-17RA with a CC′ loop decoy peptide significantly attenuated IL-17-A and IL-17E-associated inflammation [130]. These observations suggest the potential of blocking Act1 for management of IL-17-induced autoimmune diseases. Moreover, crucial transcription factors such as NF-*κ*B, C/EBP, and AP-1 [131] involved in regulating IL-17 signaling events deserve more attention and further investigation as the next generation of efficacious therapies targets.

## 5. Conclusions

IL-17 family has been shown to play active and essential roles in the pathogenesis of AILD by controlling numerous immune mediators and cellular events. Therefore, blocking IL-17-induced responses at an early stage should prevent these inflammatory events to occur or exacerbate. The discovery of IL-17 target therapy has opened up new areas of potential clinical treatment, with several clinical trials showing encouraging results. However, given the complexity of immunological interactions, more intensive investigation and cautious reflection are required. Nevertheless, there are still many key questions remaining with regard to other members of IL-17 family besides IL-17A and IL-17F. Of noted importance is that inhibition of IL-17, similar to blockade of TNF-*α* cytokine, could raise the potential risks of serious infection and other unexpected conditions. Therefore, the optimal blockade place of IL-17 pathway should properly balance therapeutic benefits and side effects in human autoimmune disease management.

## Figures and Tables

**Table 1:** Pathophysiological findings on IL-17 pathway in the development of AILD.

Disease type	Sources	Main findings	References
AIH	Patients	Elevated levels of IL-17 and IL-23 in serum. Increased Th17 cells in peripheral blood	[53, 54]
		Infiltration of Th17 cells with enhanced ROR-*γ*t expression in liver	[53]
		Increased expression of IL-17-related cytokines (IL-23, IL-21, IL-1*β*, and IL-6) in liver	[53]
		Impairment of CD39^pos^Tregs to suppress IL-17 pathway	[55]
		Greater proportions of IL-17^+^ and ROR*γ* ^+^ cells in ngTregs	[56]
	Mouse models	Elevated IL-17 levels in liver and serum	[54]
		Blockade of IL-17 attenuate inflammatory liver injury	[54]

PBC	Patients	Elevated levels of IL-17-related cytokines (IL-17, IL-23, IL-1*β*, and IL-6) and enhanced gene expression of IL-17-mediated signalling pathway (IL-23 p19, IL-23R, and IL-17) in serum	[57, 58]
		Expansion of Th17 cells with enhanced ROR-*γ*t expression in peripheral blood	[57]
		Aggregation of Th17 cells around interlobular bile ducts	[59–61]
		Enhanced expression of Th17-related cytokines and their cognate receptors (IL-23p19, IL-23p40, IL-17, and IL-23R) in liver	[61]
		Induction of inflammatory cytokines and chemokines (IL-6, IL-1*β*, IL-23p19, CXCL1, CXCL2, CXCL3, CXCL6, CXCL8, CCL2, and CCL20) by IL-17 in human BECs	[60]
		Upregulation of MIP-3*α* in human BECs for further recruit of LCs	[62]
	Mouse models		
	IL-12R*α*−/− mice	Increased frequencies of IL-17 producing cells in liver	[63]
		Induction of IL-17 responses by splenic CD4^+^ T cells cocultured with NPCs	[59]
	2OA-BSA-immunized mice	Reduction of biliary damage in IL-17A−/− mice and IL-22−/− mice. Lower levels of AMA in IL-17A−/− mice	[64]
	dnTGF*β*RII mice	Lower titres of anti-gp210 antibodies in mice with deletions of IL-17-related cytokines (IL-12p40, IL-23p19, IL-17, IL-6, and TNF-*α*)	[65]

PSC	Patients	Infiltration of IL-17^+^ lymphocytes around damaged bile ducts and in areas of neoductular proliferation	[66]
		Increased frequencies of Th17 cells by stimulation of pathogen in peripheral blood	[67]
		Induction of Th17 cells by the selective stimulation of TLR 5 and TLR 7	[66]
	Mouse models (BDL)	Elevated levels of IL-17A in serum	[68]
		Increased gene levels of IL-17-related cytokines and receptors (IL-17 A, IL-17F, IL-17RA, and IL-17RC) in liver	[68]
		Reduction of BDL-induced liver fibrosis in IL-17RA−/− mice	[68]
		Strong expression of IL-17-related cytokines and their receptors in liver resident cells (Kupffer cells, HSC)	[68, 69]
		Induction of TNF-*α* and TGF-*β* in Kupffer cells by IL-17 and in turn promotion of IL-17 expressing cells differentiation	[68, 69]

AIH: autoimmune hepatitis; PBC: primary biliary cirrhosis; BEC: biliary epithelial cells; MIP-3*α*: macrophage inflammatory protein-3*α*; LC: Langerhans cells; NPCs: nonparenchymal cells; AMA: anti-mitochondrial antibodies; dnTGF*β*RII: dominant-negative TGF-*β* receptor II; PSC: primary sclerosing cholangitis; TLR: Toll-like receptor; BDL: bile duct ligation; HSC; hepatic stellate cells; 2OA: 2-octynoic acid.

## Abbreviations

AILD: Autoimmune liver diseases
AIH: Autoimmune hepatitis
PBC: Primary biliary cirrhosis
PSC: Primary sclerosing cholangitis
CHB: Chronic hepatitis B
MS: Multiple sclerosis
RA: Rheumatoid arthritis
NK: Natural killer
DCs: Dendritic cells
Lti: Lymph tissue inducer
BEC: Biliary epithelial cells
MIP-3*α*: Macrophage inflammatory protein-3*α*
LC: Langerhans cells
NPCs: Nonparenchymal cells
AMA: Anti-mitochondrial antibodies
dnTGF*β*RII: Dominant-negative TGF-*β* receptor II
TLR: Toll-like receptor
BDL: Bile duct ligation
HSC: Hepatic stellate cells
2OA: 2-Octynoic acid
TRAF6: TNF receptor associated factor 6
NF-*κ*B: Nuclear factor-*κ*B
IBD: Inflammatory bowel disease
SLE: Systemic lupus erythematosus.
